# Effective and Stable Senomorphic Apigenin Delivery System Obtained by Supercritical Carbon Dioxide Processing

**DOI:** 10.3390/ijms26178126

**Published:** 2025-08-22

**Authors:** Anna Stasiłowicz-Krzemień, Natalia Rosiak, Giuseppe Francesco Racaniello, Nunzio Denora, Judyta Cielecka-Piontek

**Affiliations:** 1 Department of Pharmacognosy and Biomaterials, Poznan University of Medical Sciences, Rokietnicka 3 Str., 60-806 Poznan, Poland; astasilowicz@ump.edu.pl (A.S.-K.); nrosiak@ump.edu.pl (N.R.); 2 Department of Pharmacy—Pharmaceutical Sciences, University of Bari Aldo Moro, Orabona St. 4, 70125 Bari, Italy; giuseppe.racaniello@uniba.it (G.F.R.); nunzio.denora@uniba.it (N.D.); 3 Department of Pharmacology and Phytochemistry, Institute of Natural Fibres and Medicinal Plants, Wojska Polskiego 71b, 60-630 Poznan, Poland

**Keywords:** apigenin, senomorphic, neuroprotection, amorphization, supercritical conditions, solubility

## Abstract

Apigenin (AP) is a natural flavonoid with senomorphic potential and neuroprotective action; however, poor aqueous solubility (<1 μg/mL) limits its bioavailability and therapeutic use. Therefore, the aim of this study was to obtain an amorphous dispersion of AP and evaluate its biological properties. Screening of AP solubilization capabilities under supercritical carbon dioxide processing conditions showed that the system with Soluplus (SOL) achieved the greatest improvement in AP dissolution (6455.4 ± 27.2 μg/mL). Using optimized process parameters (50 °C, 6500 PSI), the AP solubility increased to 8050.2 ± 35.1 μg/mL. X-ray powder diffraction (XRPD) confirmed amorphization, aligning with improved dissolution of AP in both acidic and neutral pH media. As a result, using the PAMPA model, an improvement in AP penetration through membranes simulating gastrointestinal and blood–brain barriers was demonstrated. The significant stability of the obtained amorphous AP dispersion (12 months at room conditions) was associated with stabilizing AP–solubilizer intermolecular interactions, mainly expressed as the shifts in the bands of AP in the range of 1018–1269 cm^−1^ observed in ATR-FT-IR spectra. Chromatographic analysis confirmed the lack of AP decomposition immediately after the preparation of the amorphous dispersion, as well as after 12 months. As expected, the improvement of AP solubility is correlated with better biological activity assessed in selected in vitro tests such as antioxidant properties (2,2-diphenyl-1-picrylhydrazyl (DPPH), 2,2′-azino-bis(3-ethylbenzothiazoline-6-sulfonic acid) (ABTS), and cupric ion reducing antioxidant capacity (CUPRAC) assays) and anticholinesterase inhibition capabilities (AChE and BChE assays). The effect of the studies on improving AP solubility under supercritical carbon dioxide processing conditions is obtaining a stable amorphous AP dispersion (up to 12 months). Regardless of the pH of the media, an improvement in AP dissolution and penetration, conditioned by the passive diffusion process, through biological membranes was noted. Moreover, a more efficient antioxidant and neuroprotective effect of AP in the developed amorphous dispersion can also be suggested.

## 1. Introduction

The global population is aging rapidly, leading to a growing prevalence of age-related diseases and placing significant strain on healthcare systems [1]. Aging is associated with progressive functional decline, chronic inflammation, and increased susceptibility to degenerative conditions. A key contributor to these processes is the accumulation of senescent cells, which have irreversibly exited the cell cycle and adopt a distinct secretory profile known as the senescence-associated secretory phenotype (SASP) [2].

Senescent cells play a dual role. While they prevent the proliferation of damaged cells, their chronic presence contributes to tissue dysfunction, inflammation, and disease progression [3]. Strategies to target these cells fall into two categories: senolytics, which selectively eliminate senescent cells, and senomorphics, which modulate their harmful secretory phenotype without inducing cell death [4]. Natural compounds have shown potential as both senolytics and senomorphics, offering promising avenues for modulating cellular senescence [5,6]. Most studies investigating these natural agents have so far been conducted on cellular models [7]. One of the major challenges in translating these findings into clinical applications is improving the bioavailability of such compounds, which often exhibit poor solubility and limited systemic absorption [8,9,10,11].

Apigenin (AP) is a natural flavone found in various fruits, vegetables, and medicinal plants such as celery, parsley, chamomile, and chrysanthemum [12]. AP has recently gained recognition for its senomorphic potential, referring to its ability to modulate the phenotype of senescent cells without necessarily inducing their death. Cellular senescence, a hallmark of aging and age-related diseases, is characterized by the SASP, which promotes chronic inflammation and tissue dysfunction. AP has been identified as a compound capable of attenuating SASP, thus mitigating the harmful paracrine effects of senescent cells [13,14,15]. For instance, research by Zhang et al. has shown that AP significantly downregulates the expression of SASP factors by interfering with the interaction between HSPA8 and key signaling kinases such as ATM and p38MAPK [13,14]. Additional findings by Martínez-Gutiérrez et al. reveal that AP, in combination with phloretin, effectively modulates SASP in senescent fibroblasts, thereby enhancing its senomorphic profile [16]. Moreover, AP reduces markers of senescence like p21 and p16 in various cell types, including osteoblasts and bone marrow stromal stem cells, suggesting its relevance across multiple tissues [17]. When used alongside established senolytic agents, AP may offer synergistic benefits by targeting both senescent cells and their pro-inflammatory secretions [14,16]. Its senomorphic capacity is further supported by its antioxidative functions, as it reduces reactive oxygen species (ROS) and alleviates oxidative stress, both key contributors to the onset and maintenance of cellular senescence [18,19]. Furthermore, evidence supports the role of the AMPK signaling pathway in mediating AP protective effects against age-related cellular dysfunction [20]. Together, these multifaceted mechanisms highlight AP as a promising candidate in the development of geroprotective therapies [21]. It also exhibits a broad spectrum of biological activities, including anti-inflammatory, antioxidant, anticancer, and neuroprotective effects. It has shown the ability to reduce UVA-induced cytotoxicity and modulate key processes such as oxidative stress, apoptosis, inflammation, and neurodegeneration [22,23,24]. It mitigates neuroinflammation, neuronal excitability, and apoptosis in neurodegenerative disease models [25], and has shown protective activity in PC12 cells, including against 1-methyl-4-phenylpyridinium ion-induced neurotoxicity [26]. AP has also been reported to inhibit amyloid-β (Aβ) aggregation and activate tropomyosin receptor kinase B (TRKB) signaling, mechanisms that are crucial in the pathogenesis of Alzheimer’s disease [27]. Furthermore, it has demonstrated protective effects in cerebral ischemia/reperfusion injury models [28,29], along with its ability to scavenge superoxide anions and enhance antioxidative enzyme activity [30]. The link between aging, oxidative stress, and neurodegeneration is well established, with reactive oxygen species (ROS) playing a central role in cellular aging and damage. AP’s potent antioxidant properties and ability to modulate ROS levels further support its relevance in geroprotective strategies. However, AP exhibits very low solubility in water, which may impact its bioavailability and therapeutic efficacy [31].

Various methods could be employed to enhance the solubility of a compound. These include the formation of amorphous solid dispersions [32], cocrystal formation [33], preparation of a salt form of the compound [34], micronisation or nanonisation techniques [35,36], as well as the use of surfactant-based counterions to enhance water solubility [37]. Other strategies have also been reported, such as glycoconjugate formation, particularly relevant for flavonoids [38,39], and polymorph screening to identify crystal forms with superior dissolution properties [40,41]. Among methods enabling amorphization of a compound, there are spray drying, hot melt extrusion, electrospinning, ball milling, and freeze-drying [42,43,44]. It has also been established that an increase in the solubility of poorly soluble AP is effectively correlated with an enhancement of its oral bioavailability [45,46]. Amorphization increases surface area and disrupts the ordered crystalline lattice, thereby weakening intermolecular forces and promoting stronger interactions with solvents, which leads to improved dissolution kinetics and overall solubility enhancement [47,48,49]. In addition to improving solubility, amorphization can also modify the drug release profile, enabling either rapid release for immediate therapeutic effect or controlled release, depending on the formulation design [50].

From a processing perspective, the degree of crystallinity can significantly influence formulation steps. Crystalline powders, due to their regular particle morphology and lower interparticle cohesion, generally exhibit superior flowability but may be more prone to segregation during blending. In contrast, amorphous powders often display higher cohesion and irregular particle shapes, which can impair flow but, when properly dispersed, may facilitate more homogeneous mixing [51,52,53]. While amorphous materials may exhibit reduced flowability, they often possess greater compressibility [54]. However, mechanical stress during compression can induce partial crystallization in amorphous formulations [55]. It is therefore critical to optimize processing conditions, as excessive heat or prolonged processing times can cause both degradation and an undesired increase in crystallinity [56].

Processes such as hot-melt extrusion and spray drying are commonly employed to produce amorphous solid dispersions; however, they require elevated processing temperatures, which may pose a risk of thermal degradation for heat-sensitive compounds [57]. An alternative strategy for inducing amorphization is the use of supercritical carbon dioxide (scCO_2_), which enables the transformation of a compound into its amorphous form under milder thermal conditions [58]. Owing to its unique physicochemical properties, scCO_2_ processing offers a mild, energy-efficient, and environmentally friendly approach that allows amorphization at low temperatures without the need for toxic organic solvents, while also facilitating the incorporation of solubility-enhancing excipients.

AP has been classified as a BCS Class II compound due to its high permeability but low solubility, posing significant challenges for achieving optimal bioavailability [59]. The poor solubility of AP limits its absorption and therapeutic potential. Various formulation approaches have been investigated to address this limitation. For instance, AP–phospholipid phytosomes have been developed and characterized, showing promising improvements in solubility, in vivo bioavailability, and antioxidant activity [60]. Nanotechnology-based delivery systems, such as nanoparticles prepared via liquid antisolvent precipitation, have demonstrated enhanced solubility and the potential for controlled drug release [61]. Self-microemulsifying drug delivery systems have been reported to markedly increase AP solubility and improve dissolution profiles [62]. Furthermore, hot-melt extrusion has emerged as an effective solid-dispersion technique for enhancing AP’s solubility and dissolution rate [63].

In the present study, the objective was to overcome the solubility limitations of AP by developing an amorphous solid dispersion using solubility-enhancing excipients. This approach was inspired by the promising results obtained in our previous work [64]. Supercritical carbon dioxide processing was employed to induce amorphization under mild thermal conditions, which is in line with sustainable and energy-efficient practices. AP is known for its senomorphic effects; however, its role in promoting longevity likely involves broader mechanisms. Beyond modulating SASP, AP influences neuroinflammation, oxidative stress, and cholinergic signaling, key factors in aging-related CNS and inflammatory dysfunction [31,65,66,67]. Enhancing its solubility is therefore crucial to unlocking its full pleiotropic potential. To assess whether improved solubility translates into greater biological activity, the delivery system was tested in vitro for antioxidant capacity (DPPH, ABTS, CUPRAC) and anticholinesterase activity (AChE, BChE).

## 2. Results

### 2.1. Preparation of the Systems and Solubility Studies

The solubility of AP was evaluated in the presence of various carriers. The data reveal a striking difference in the solubility of AP when formulated with Soluplus (SOL) compared to other carriers (Table 1). While AP alone exhibits a solubility of less than 1 μg/mL, SOL markedly enhances its solubility to 6455.4 ± 27.2 μg/mL, representing an extraordinarily high increase. This dramatic improvement in solubility suggests that SOL plays a key role in overcoming AP’s inherent poor solubility. In contrast, other excipients increased the solubility only to levels ranging from 1.0 to 10.4 μg/mL, highlighting the superior AP solubilizing capability of SOL.

**Figure 1:**
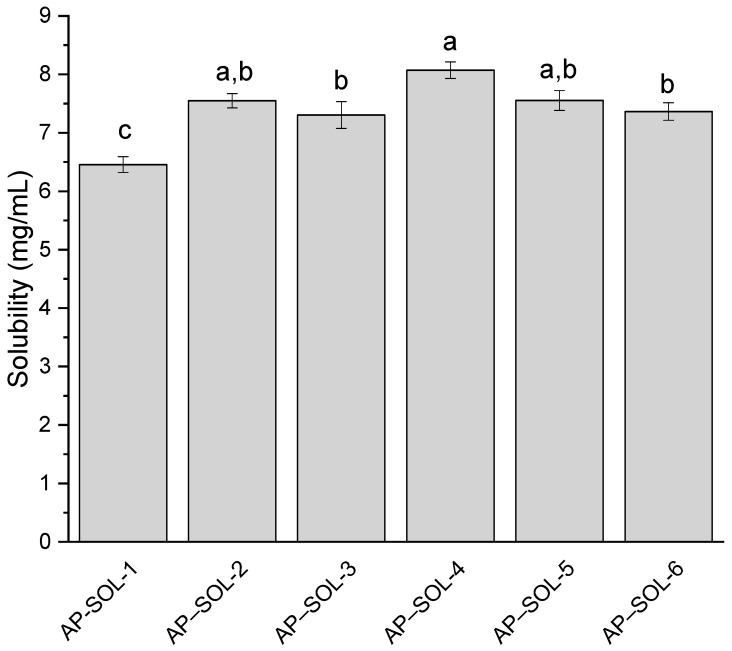
The solubility of apigenin (AP) in the delivery systems with Soluplus (SOL) obtained at 50 °C, 65 °C, and 80 °C, and under 5000 PSI and 6500 PSI (AP–SOL 1–6). Bars not sharing the same letter (a–c) are significantly different at *p* < 0.05, as indicated by the Compact Letter Display.

After selecting the carrier, process parameters were optimized (Figure 1). The solubility of AP was evaluated at temperatures of 50 °C, 65 °C, and 80 °C under two pressure conditions: 5000 PSI and 6500 PSI (AP–SOL 1–6). Results showed that solubility increased or remained statistically similar with pressure, with the highest value observed in AP–SOL 4 (8.07 mg/mL). At both pressure levels, solubility peaked or remained statistically similar with increasing temperature, indicating a temperature-dependent solubility profile. The optimal condition for maximum AP solubility was determined to be 50 °C at 6500 PSI.

### 2.2. X-Ray Powder Diffraction

**Figure 2:**
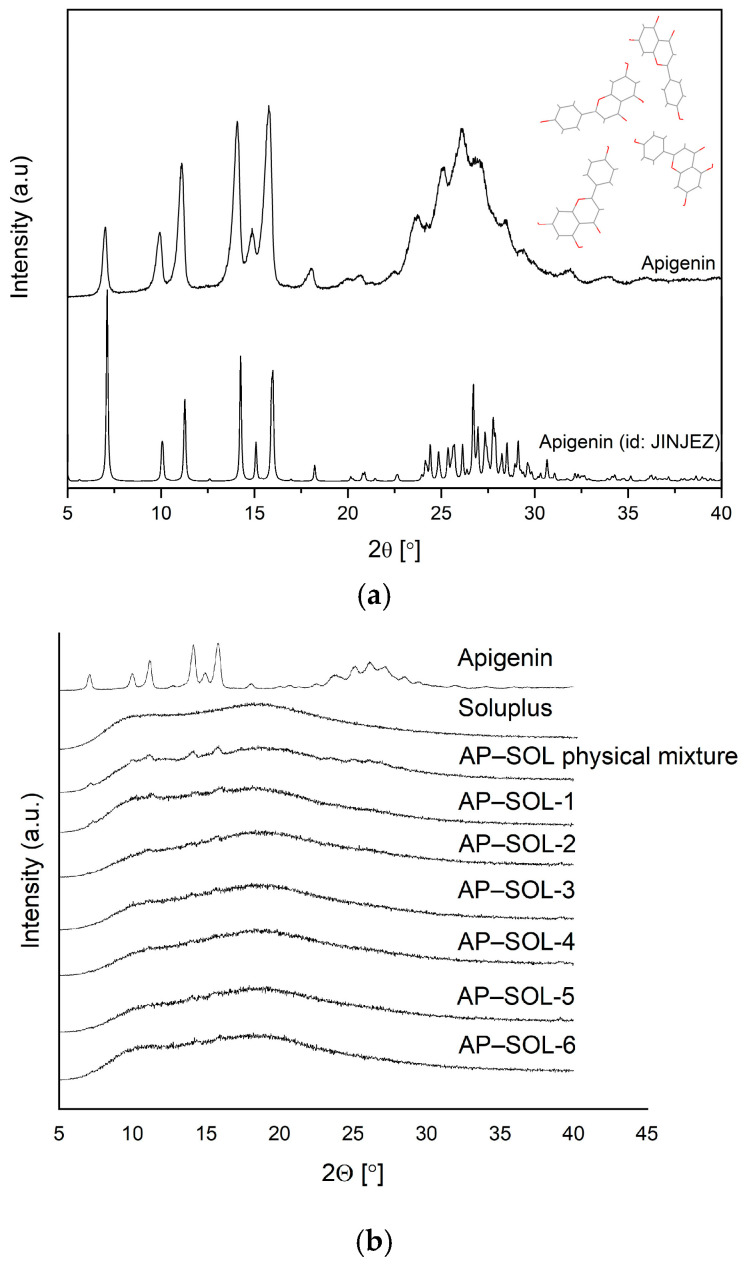
(**a**) Crystalline structure of AP (red color is the oxygen atom) and calculated powder pattern based on the crystalline structure of AP (CCDC id: JINJEZ and deposition number: 2232365) compared with the experimental diffraction pattern of AP; (**b**) The XRPD patterns of apigenin (AP), Soluplus (SOL), their physical mixture and the delivery systems of AP and SOL obtained at 50 °C, 65 °C, and 80 °C, and under 5000 PSI and 6500 PSI (AP–SOL 1–6).

The XRPD patterns confirmed the crystalline nature of AP, as evidenced by the presence of sharp and intense Bragg peaks. To support phase identification, a theoretical powder diffraction pattern was generated from the CIF obtained from the Cambridge Crystallographic Data Centre (CCDC 2232365; accessed on 31 July 2025) (Figure 2a). The experimental diffraction pattern showed excellent agreement with the simulated data, particularly in the region of the most characteristic reflections (range 6–19°2θ)—previously reported in the literature [68] as characteristic of AP—clearly observed in both theoretical and experimental patterns, further confirming the identity and crystallinity of the sample. The recorded AP diffraction pattern is consistent with those published previously [68,69], and the SOL diffraction pattern exhibited a typical amorphous halo (Figure 2b).

In the physical mixture, the Bragg peaks of AP remained clearly visible, indicating the presence of a crystalline form of AP. In contrast, the delivery systems, particularly the one prepared at 50 °C and 6500 PSI, which also showed the highest increase in solubility, displayed a broad amorphous halo with a marked reduction or complete disappearance of the Bragg peaks. This suggests successful amorphization of AP under these processing conditions, contributing to its enhanced solubility. Based on the 24 h solubility study and XRPD analysis, the system obtained at 50 °C and 6500 PSI (AP–SOL-4) was selected as the optimal one, as it exhibited the highest increase in solubility. From this point forward, the sample AP–SOL-4 will be referred to as the “AP–SOL system”. This correlates with the formation of an amorphous structure, as indicated by the disappearance of Bragg peaks in the XRPD pattern. Notably, these conditions proved more effective than those at higher temperatures, suggesting that lower thermal input combined with higher pressure is more favorable for inducing amorphization, likely due to reduced energy barriers. Further studies of in vitro biological activity, apparent solubility, permeability, and physical stability were performed for this delivery system.

### 2.3. Fourier-Transform Infrared Spectroscopy

**Figure 3:**
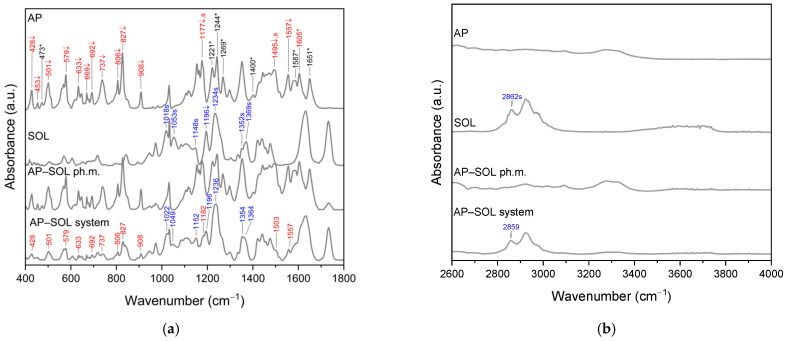
FT-IR analysis: apigenin (AP), Soluplus (SOL), physical mixture of AP and SOL, and their delivery system in the range 400–1800 cm^−1^ (**a**) and 2600–4000 cm^−1^ (**b**). Legend: band disappearance (*); intensity decrease (↓); band shift (s); black values are AP’s peaks not observed in the system spectrum, whereas red values are AP’s peaks, and blue values are SOL’s peaks for which changes are observed in the system spectra.

FTIR spectroscopy was used to analyze the interactions responsible for the formation of bonds between AP and SOL. The spectra of AP, SOL, the physical mixture, and the system were recorded (Figure 3). The spectra of the physical mixtures reveal signals attributable to both AP and SOL, suggesting that no chemical interaction takes place between them. Meanwhile, the AP–SOL system shows changes in characteristic bands assigned to AP and SOL. To facilitate the interpretation of these changes, specific symbols were marked on the spectra next to the relevant AP’s (red value) and SOL’s (blue value) bands: s denotes a shift in band position, ↓ indicates a decrease in intensity, and * means that the peak was not observed in the system and observed in the physical mixture. These symbols refer to changes observed in the system compared to the individual components (AP, SOL). Detailed descriptions of the changes are summarized in Table 2.

In the spectrum of pure AP, a number of bands were observed, which were assigned to torsional and bending vibrations of C–C, C–O bonds, and hydroxyl groups. The full interpretation of the AP bands was described in Rosiak et al. [64]. The bands exhibiting changes in AP–SOL, which provide evidence for the formation of interactions between AP and SOL, will be presented in this article.

For example, in AP spectra, the bands in the range of 428–908 cm^−1^ were mainly assigned to torsional vibrations of HCCC and out-of-plane vibrations of OCCC. These bands are observed in the physical mixture and not observed in the spectrum of the AP–SOL system (Figure 3a, symbol *). Next, the range of 1352–1495 cm^−1^ corresponds to the bending vibrations of HCC and stretching CC vibrations, while the bands at 1587, 1605, and 1651 cm^−1^ are assigned to the CC and OC stretching vibrations. In this range for AP–SOL, no AP bands are observed, which for the pure compound were recorded at 1400 cm^−1^, 1587 cm^−1^, and 1651 cm^−1^; moreover, the bands at 1495 cm^−1^, 1557 cm^−1^, and 1605 cm^−1^ have lower intensity, while the band at 1605 cm^−1^ is visible as shoulder of SOL’s peak observed at 1632 cm^−1^.

In the range of 1018–1369 cm^−1^, the presence of SOL’s bands was mainly assigned to bending vibrations of HCC, HOC, and stretching vibrations of COC in ether groups, whereas the band at about 2862 cm^−1^ corresponds to the CH stretching vibrations. In the AP–SOL system, slight shifts in these bands were observed. The shifts in the characteristic SOL’s bands: 1369 → 1364 (O(C)O or NH) and 2862 → 2859 (νCH), and AP’s bands: 1177 → 1182 (δHOC + δHCC), 1495 → 1503 (δHCC + νCC) as well as the decrease in intensity and the absence of peaks (present in ph.m.) indicate the formation of hydrogen bonds between AP and SOL. The bonds are probably formed between the NH or CH group of SOL and the CO group of AP. In addition, the broadening of the 1632 band (SOL, C=O s) in tertiary amide in the caprolactam [74,75,76] or C(O)N [77] may be related to the change in vibration energy resulting from the formation of hydrogen bonds between the C=O group of SOL and the hydroxyl or carbonyl group of AP. The results indicate that hydrogen bonding contributes to the stabilization of the amorphous state of AP, as evidenced by XRPD data. These findings are consistent with previous studies [78,79,80], suggesting that the non-observation of shift in vibrational peaks typically results from crystal structure changes (i.e., transition to an amorphous form) or the formation of intermolecular hydrogen bonds. For instance, Lu et al. [81] confirmed the formation of hydrogen bonds involving the C=O group of SOL in amorphous dispersions of felodipine.

### 2.4. The Dissolution-Rate Studies

**Figure 4:**
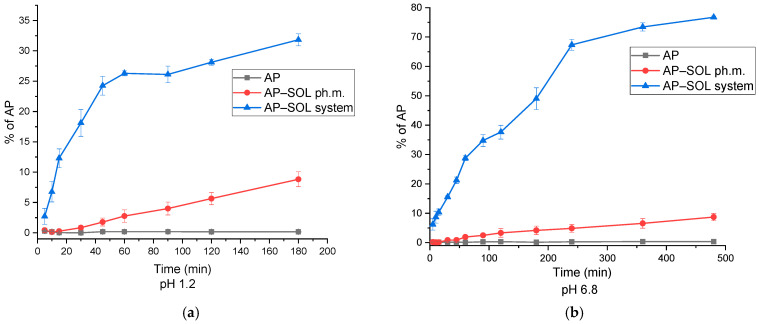
The dissolution profiles of pure apigenin (AP), a physical mixture of apigenin with Soluplus (AP–SOL ph.m.), and a Soluplus-based delivery system (AP–SOL system) evaluated in media simulating gastric (pH 1.2 (**a**)) and intestinal (pH 6.8 (**b**)) environments.

At pH 1.2, pure AP exhibited minimal release throughout the study, indicating poor solubility in acidic conditions (Figure 4). The physical mixture showed an improved release, with a gradual increase over time, reaching approximately 8.8% at 180 min. In contrast, the SOL delivery system significantly enhanced AP release, achieving 31.8% at 180 min, which correlates with the amorphous transformation observed in the XRPD data, particularly under optimized processing conditions. At pH 6.8, a similar trend was observed. Pure AP again showed low release values; the physical mixture achieved slightly higher release, with a maximum of 8.7%. The delivery system demonstrated the most pronounced enhancement, reaching a release of 76.71%, confirming its superior performance in both gastric and intestinal environments. These findings indicate that the delivery system not only improves solubility but also provides sustained and enhanced release of AP, independent of pH. The delivery system outperformed both the pure AP and the physical mixture across all conditions, highlighting the effectiveness of the system with SOL in enhancing the apparent solubility of AP.

### 2.5. In Vitro Parallel Artificial Membrane Permeability Assay

**Figure 5:**
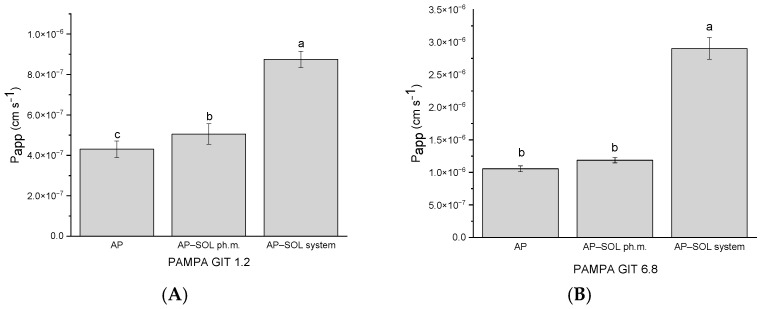
Apparent permeability coefficient values of apigenin (AP), Soluplus (SOL), physical mixture of AP and Soluplus (SOL), and their delivery system in PAMPA study in pH 1.2 (**A**) and 6.8 (**B**). Bars not sharing the same letter (a–c) are significantly different at *p* < 0.05, as indicated by the Compact Letter Display.

The Papp of the AP was evaluated using the PAMPA at pH 1.2 and 6.8, simulating gastrointestinal conditions (Figure 5). At pH 1.2, all tested samples fell within the range of moderate permeability. A slight increase in AP permeability was observed with the physical mixture, while the AP–SOL system demonstrated the greatest increase to 8.75 × 10^−7^ cm/s. Although the permeability remained in the moderate range, a clear enhancement was seen with increasing degrees of solubilization. At pH 6.8, the permeability of the AP alone reached 1.05 × 10^−6 ^ cm/s, classifying it as highly permeable [82]. The physical mixture showed a slightly higher Papp, while the system exhibited a significant increase to 2.90 × 10^−6^ cm/s. All samples at this pH level were thus considered highly permeable, with the delivery system demonstrating the most pronounced improvement.

**Figure 6:**
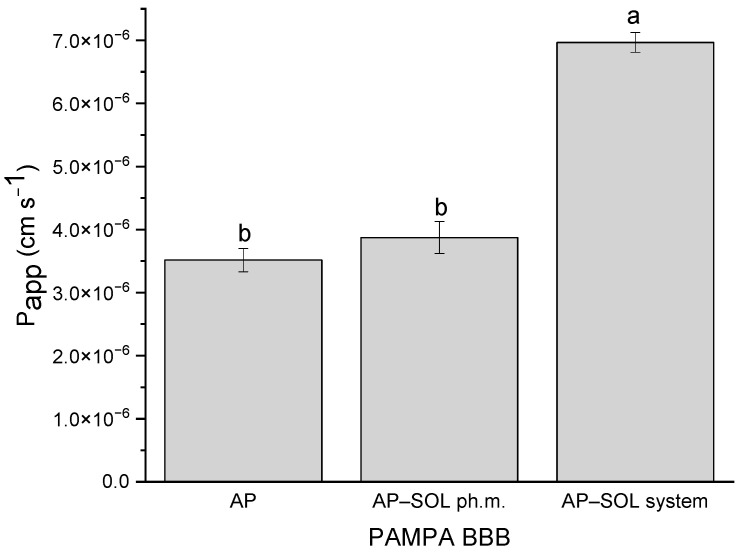
Apparent permeability coefficient values of apigenin (AP), Soluplus (SOL), physical mixture of AP and Soluplus (SOL), and their delivery system in the PAMPA study in the blood–brain barrier (BBB) assay. Bars not sharing the same letter (a, b) are significantly different at *p* < 0.05, as indicated by the Compact Letter Display.

In the PAMPA-BBB model simulating passive diffusion across the blood–brain barrier, AP alone exhibited a Papp of 3.51 × 10^−6^ cm/s, placing it in the medium permeable category (Figure 6). It was only the delivery system with Sol that increased the permeability statistically significantly, reaching 6.97 × 10^−6^ cm/s, reaching a high permeability threshold.

### 2.6. Stability Studies

**Figure 7:**
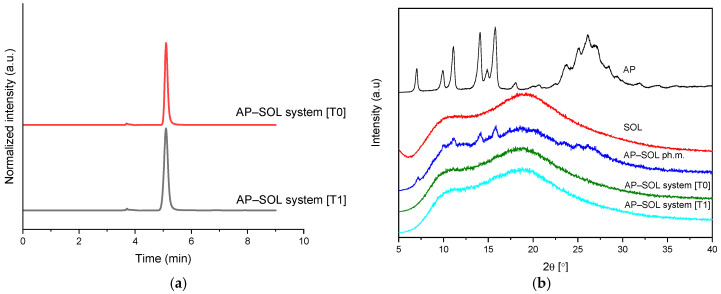
The chemical (**a**) and physical (**b**) stability of apigenin (AP) solid dispersion with Soluplus (SOL)—directly after preparation (T0) and after 12 months storage in ambient conditions (T1).

HPLC analysis confirmed the chemical stability of AP within the amorphous dispersion, as no new peaks or changes were observed in the chromatographic profiles between the initial (T0) and 12–month (T1) samples, demonstrating the absence of degradation products (Figure 7).

Concurrently, the XRPD results at the 12-month time point (T1) continued to display an amorphous diffraction pattern, indicating that the sample maintained its amorphous character throughout the storage period. These combined results indicate that the amorphous solid dispersion of the AP remained both chemically stable and structurally amorphous after 12 months of storage under ambient conditions.

### 2.7. Antioxidant Activity

The antioxidant activity of AP, its physical mixture with SOL, and the amorphous delivery system was evaluated using DPPH, ABTS, and CUPRAC assays with the samples used after the solubility study (Table 3) (AP concentrations: <1 μg/mL for AP, 0.93 mg/mL for the physical mixture, 8.05 mg/mL for the delivery system). AP exhibited poor antioxidant activity in all assays (DPPH and ABTS < 0.1%; CUPRAC < 0.01), due to its poor solubility. In contrast, both physical mixture and the delivery system improved antioxidant activity. The physical mixture showed moderate effects (DPPH: 12.3 ± 0.9% (18.4 mg Trolox/g), ABTS: 85.2 ± 2.4%, CUPRAC: 0.51 ± 0.03 (62.3 mg Trolox/g)), while the solubility-enhanced system demonstrated the highest antioxidant capacity (DPPH: 27.9 ± 0.7% (6.1 mg Trolox/g), ABTS: 84.9 ± 3.1%, CUPRAC: >1.00), with statistically significant improvements across all assays compared to pure AP (*p* < 0.05). It is worth noting that the results for the delivery system in ABTS and CUPRAC are in the plateau area. The IC_50_/IC_0_._5_ values were determined for AP using DMSO as the solvent. The values were 27.7 ± 0.6 mg/mL (DPPH), 59.0 ± 3.1 μg/mL (ABTS), and 0.91 ± 0.02 mg/mL (CUPRAC). These results confirm that improving AP’s solubility significantly enhances its observable antioxidant activity.

### 2.8. Anticholinesterase Activity

**Figure 8:**
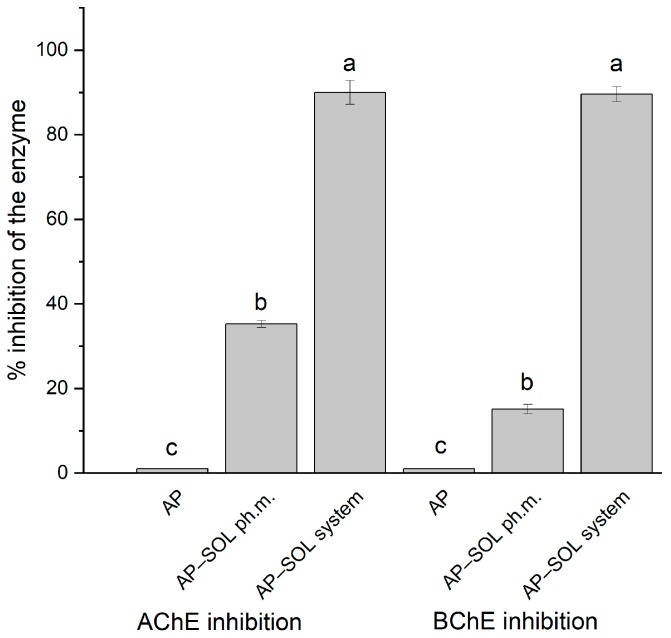
The potential of the apigenin (AP), physical mixture (ph.m.), and the delivery system with Soluplus (SOL) to inhibit acetylcholinesterase (AChE) and butyrylcholinesterase (BChE). Bars not sharing the same letter (a–c) are significantly different at *p* < 0.05, as indicated by the Compact Letter Display.

AChE inhibition increased substantially from below 1.0% for the pure AP to 35.2% in the physical mixture and 90.0% in the delivery system (Figure 8), highlighting a strong enhancement due to improved solubility as the samples after solubility study were used (<1 μg/mL for AP, 0.93 mg/mL for the physical mixture, 8.05 mg/mL for the delivery system). Similarly, BChE inhibition rose from below 1.0% to 15.1% and 89.6%, respectively, confirming that both enzyme targets benefit significantly from the optimized delivery approach. The values for the delivery systems fall within the plateau region of the curve, as the IC_50_ values obtained for the AP in DMSO are 1.6 mg/mL for AChE and 4.3 mg/mL for BChE, which are higher values than those observed for galantamine (17.4 μg/mL and 155.3 μg/mL, respectively).

## 3. Discussion

AP is a natural flavonoid whose biological properties are limited due to its poor solubility, ~0.6 μg/mL −2.16 μg/mL [31,60,83]. Within this study, the solubility was assessed as <1 μg/mL (~0.2 μg/mL). Discrepancies in solubility values are common for natural compounds and may result from factors related to the studied substance, such as differences in extraction and purification procedures, the origin of the plant material, the degree of purity, as well as methodological conditions during the study, including incubation time, pH, and temperature. The solubility in organic solvents is increased (0.001–1.63 mg/mL) [83]. The AP solubility was also studied in phosphate buffers ranging from pH 1.0 to 7.5, in a 37 °C water bath with shaking over 3 days. Indeed, pH has been shown to impact AP solubility, with previous studies reporting values ranging from 1.01 μg/mL at lower pH to 2.16 μg/mL at pH 7.5 after 72 h of incubation at 37 °C [84]. In the current study, the carrier that showed the biggest increase in AP solubility was SOL. Under optimal conditions, the amorphous system of AP with SOL was obtained, which increased the water solubility of AP to 8.05 mg/mL, representing more than an 8000-fold improvement. According to the European Pharmacopoeia, substances are classified into solubility categories based on the number of parts of solvent required to dissolve 1 part of solute: very soluble is less than 1 part of solvent to 1 part of solute (greater than 1000 g/L); freely soluble is 1 to 10 parts of solvent to 1 part of solute (100–1000 g/L); soluble is 10 to 30 parts of solvent to 1 part of solute (33.3–100 g/L); sparingly soluble is 30 to 100 parts of solvent to 1 part of solute (10–33.3 g/L); slightly soluble is 100 to 1000 parts of solvent to 1 part of solute (1–10 g/L); and insoluble is more than 10,000 parts of solvent to 1 part of solute (less than 0.1 g/L) [85]. For AP, the solubility improved from less than 1 μg/mL to soluble, reaching 8050.2 ± 35.1 μg/mL, demonstrating a significant increase in solubility. This degree of enhancement exceeds those reported in many other solubility-enhancing strategies. Zhang et al. prepared binary mixed micelles system of AP with SOL/Pluronic F127 by ethanol thin-film hydration method; they managed to obtain a 3442-fold increase in AP solubility to 5.61 mg/mL in water [86]. In another study, a solid dispersion of AP with mesoporous silica nanoparticles was prepared using a physical absorption method. The increased solubility studied in a 0.4% solution of sodium dodecyl sulfate over a 48 h period was measured at 25.11 µg/mL [87]. Wu et al. assessed AP as insoluble in water and slightly soluble in ethanol (1.93 mg/mL), and prepared an inclusion complex of AP with 2-hydroxypropyl-β-cyclodextrin using the liquid antisolvent precipitation and solvent removal combination methods [88]. Solubility of the complex and AP in artificial gastric juice containing 0.4% Tween-80 and artificial intestinal juice at 37 °C after 48 h was assessed as 13.06 μg/mL and 0.19 μg/mL in acidic conditions, and intestinal conditions as 6.81 μg/mL and 0.17 μg/mL, respectively. Chitosan-coated AP liposomes prepared by ethanol injection and spray drying increased AP water overnight solubility at room temperature to 10.22 ± 0.18 mg/L [89]. Telange et al. prepared an AP-phospholipid phytosome, which increased the AP water solubility from approximately 0.6 μg/mL to over 35-fold in the aqueous solubility at room temperature for 24 h [60].

In the FT-IR spectrum of pure AP in the range of 428–908 cm^−1^, numerous bands corresponding to torsional (τHCCC) and out-of-plane (γOCCC) vibrations associated with C–C, C–O bonds, and hydroxyl groups were detected. These bands are also present in the spectrum of the AP–SOL system. This suggests that the main chemical structure of AP has not changed in the studied system. The shifts in the bands in the range of 1018–1269 cm^−1^, in particular 1177 → 1182 cm^−1^ (AP, δHOC + δHCC) and 1234 cm^−1^ → 1236 cm^−1^ (Sol, νCOC in the ether groups), and the bands in the higher ranges, indicate intermolecular interactions—such as the formation of hydrogen bonds between the hydroxyl groups of AP and the ether groups (C–O–C) present in the polymer structure. The possibility of the formation of weak hydrogen bonds of the C–H···O=C or C–H···O–C type is also indicated, in which AP acts as a donor and Sol as an acceptor. This is indicated by the shifts in the Sol (1369 cm^−1^ → 1364 cm^−1^: O(C)O or NH) and AP (2862 cm^−1^ → 2859 cm^−1^: νCH) bands. Similar observations were published in Rosiak et al. for the combination of hesperidin (Hes) with SOL (hydrogen bond between the C–H and/or O–H groups of Hes and the C–O group of Sol) and pterostilbene with Sol (hydrogen bond between the C–O, –O–H and/or –CH groups of PTR, and the O–H and C–O group of Sol) [78,90]. Lu et al. also indicate the possibility of forming hydrogen bonds with the C=O group in Sol (the case concerned amorphous dispersions of felodipine with Sol). The hydrogen bonds are responsible for maintaining the amorphous state of AP. The resulting interactions may improve the solubility of AP in water [81].

Amorphization refers to the process of converting a crystalline substance into an amorphous or non-crystalline form [91,92]. In the context of increasing solubility, amorphization can significantly enhance the dissolution rate of a substance in water. Amorphous materials lack the long-range order found in crystalline structures, resulting in a higher surface area per unit mass. This increased surface area exposes more molecules to the surrounding solvent, allowing for faster dissolution kinetics. In a crystal, molecules are neatly stacked in a pattern, like books on a shelf. This orderly arrangement makes it difficult for solvent molecules to squeeze in between the molecules. Amorphization disrupts this lattice, reducing the diffusion barrier and facilitating the penetration of solvent molecules into the material. The absence of ordered crystal structures in amorphous materials promotes closer contact between solvent molecules and solute particles. This contact enhances the interactions between the solute and the solvent, leading to faster dissolution rates. Amorphous materials typically possess higher free energy compared to their crystalline counterparts. This higher energy state makes the molecules more prone to interacting with the surrounding solvent, promoting dissolution. There are many examples in the literature of amorphous compounds showing increased solubility after amorphization [93,94,95].

Comparing dissolution results is challenging due to variations in testing conditions. Dissolution studies are conducted in different media, including those containing various concentrations of surfactants, which accelerate dissolution rates. Additionally, methods such as the paddle apparatus, baskets, and dialysis bags are employed, each yielding different results. Notably, differences are in the dissolution rate of pure AP, ranging from almost none, through a few percent, to as high as several dozen %. In Zhang et al.’s study, SOL^®^/Pluronic F127 binary mixed micelles exhibited sustained release of AP in PBS containing 1% (*w*/*v*) Tween 80 compared to free AP, as demonstrated by a slower release rate in the first 12 h; AP showed a release of 89.6% [86]. In comparison, the micelles system was 62.4%, whilst over 72 h in simulated intestinal medium, as AP reached 92.6%, and in micelles 71.9% [86]. The increased dissolution rate achieved through the mixed micelles led to a significant enhancement in oral bioavailability, resulting in a 4.03-fold increase compared to free AP in rats. In the current study, similar overall trends in pH-dependent release were observed compared to those reported by Rosiak et al., where the release study was performed in a beaker-based setup using 30 mL of medium. In both studies, higher release occurred at pH 6.8 than at pH 1.2, indicating that intestinal conditions favor the solubilization of AP regardless of the specific polymer or experimental setup used [64]. The in vitro release of AP from the inclusion complex HP-β-CD in artificial gastric juice with 0.4% Tween 80 and artificial intestinal juice in a dialysis bag was 93.71% and 74.46% at around 24 h, respectively, whilst pure AP reached 6.15% and 6.01% in the same time [88]. The bioavailability of AP was increased 3.97 times by the complex. In vitro release studies of AP from bioactive self-nanoemulsifying drug delivery system in water or with 1% sodium lauryl sulfate delivery system showed a maximum release of 89.2% of the AP compared to pure AP 31.2% in 120 min, which also increased flavonoid bioavailability [96]. Evidence from both in vitro and in vivo literature studies suggests a link between improved dissolution behavior and enhanced oral bioavailability of AP. However, further in vivo studies are needed to validate the in vitro results observed in the current study.

AP exhibits low solubility and high permeability, categorizing it as a class II drug in the BCS [97]. Intestinal absorption studies revealed concentration-dependent permeability in the duodenum and jejunum, suggesting both passive and active transport mechanisms [84]. In contrast, the ileum and colon showed concentration-independent permeability, indicating primarily passive transport. The duodenum was identified as the main absorption site for AP. Zulkifli et al. reported an apparent permeability coefficient using Caco-2 cell monolayers of less than 20 × 10^−6^ cm/s, indicating limited permeability from the apical to basolateral sides, while an efflux ratio greater than 1 suggests that AP is subject to active transport or efflux mechanisms [98]. In Zhang et al.’s study, AP demonstrated good permeability across the intestinal epithelial barrier in Across Caco-2 Cell Monolayers, whilst AP-loaded mixed micelles with SOL^®^/Pluronic F127 resulted in slightly increased permeability [86]. Moreover, the findings by Sánchez-Marzo et al. demonstrated that AP displays improved permeability when formulated in self-nanoemulsifying drug delivery systems, leading to enhanced transcellular absorption compared to its intact form [99,100]. These findings are in line with the current study, where AP demonstrated enhanced permeability under intestinal conditions, especially when using a delivery system with SOL.

In the current study, the PAMPA showed an increase in permeability with the delivery system, likely due to the higher concentration of unbound AP available for passive diffusion through micellar solubilization. However, PAMPA measures only passive transport and does not capture active uptake, efflux inhibition, or mucus permeation. Given that Soluplus can promote supersaturation, inhibit P-glycoprotein, and alter membrane properties [101,102], the in vivo permeability enhancement may exceed that observed in vitro. Although the in vitro results are promising, in vivo pharmacokinetic studies are needed to confirm them, as gastrointestinal conditions, enzymatic degradation, mucosal barriers, metabolic stability, and systemic clearance will influence overall bioavailability.

In the studies of Könczöl et al. and Sánchez-Martínez et al., AP was also confirmed to cross the blood–brain barrier using the PAMPA-BBB assay, supporting its potential for central nervous system activity [103,104]. The mechanism by which AP crosses the BBB involves significant factors, including its lipophilicity, which is a crucial determinant of many flavonoids’ ability to reach central nervous system (CNS) tissues after oral administration. According to Talebi et al., various dietary flavonoids, including AP, can effectively cross the BBB, as evidenced by their presence in the CNS following oral absorption [105]. This is consistent with the findings of Wong et al., who examined the pharmacokinetics of AP and noted that despite its lipophilic nature, challenges such as poor solubility hinder its absorption and bioavailability in the CNS [106].

In our previous studies on amorphous solid dispersions prepared with ball milling containing Pluronic^®^ F-68 and Pluronic^®^ F-127, physical changes such as partial recrystallization were observed despite preserved chemical stability [64]. In contrast, the present amorphous dispersion prepared with supercritical carbon dioxide processing with SOL demonstrates superior overall stability. Specifically, the current delivery system not only maintained chemical integrity, as confirmed by HPLC, but also retained its fully amorphous character over 12 months, as evidenced by XRPD analysis. The significant stability of the obtained amorphous AP dispersion might be associated with stabilizing AP–solubilizer intermolecular interactions, mainly expressed as the shifts in the bands of AP in the range of 1018–1269 cm^−1^ observed in ATR-FT-IR spectra. This enhanced stability underscores the improved performance of the current delivery system preparation strategy in protecting both the molecular and physical integrity of the AP under ambient storage conditions.

Enhancement of solubility is a critical factor in improving the bioavailability of poorly water-soluble natural compounds [60]. Increased solubility facilitates better dissolution in biological fluids, promoting more efficient absorption across biological membranes. This, in turn, leads to higher systemic concentrations of the active compound, enhancing its therapeutic efficacy. Poor solubility not only limits bioavailability but also compromises the biological activity of natural compounds, as insufficient solubility can hinder their interaction with cellular targets. This is particularly evident in antioxidant activity, where low solubility reduces the compound’s ability to scavenge free radicals effectively. Studies on curcumin using hot-melt extrusion to create amorphous solid dispersions have shown increases not only in solubility but also in bioactivity, including enhanced antioxidant effects [107]. The inclusion of solubilizing excipients, such as phospholipids or polysaccharides, fortifies the amorphous state and enhances the antioxidant capability of the dispersed soluble molecules [108,109]. The increase in solubility of an AP is also associated with enhanced antioxidant activity, as was presented in the current work. In the study of Ang et al., the improved aqueous solubility of AP, attributed to its encapsulation in spray-dried chitosan-coated liposomes, led to enhanced antioxidant activity studied through in vitro antioxidant assays, ABTS radical scavenging assay, the Oxygen Radical Absorbance Capacity (ORAC) assay, and the Ferric Reducing Antioxidant Power (FRAP) assay [89]. In the study of Telange et al., the enhanced aqueous solubility of AP, attributed to its incorporation into an AP-phospholipid phytosome, resulted in improved antioxidant activity by significantly increasing the levels of glutathione, superoxide dismutase, catalase, and decreasing the levels of lipid peroxidase [60]. Increased solubility of bioactive compounds not only enhances their antioxidant capacity but also facilitates a broader spectrum of biological activities, including more effective enzyme inhibition. This improved molecular interaction with enzymatic targets can significantly strengthen their therapeutic potential. For example, the encapsulation of cedar essential oil into β-cyclodextrin inclusion complexes prolonged its acetylcholinesterase inhibitory activity, whilst in the case of curcumin and piperine, the formation of amorphous systems via hot-melt extrusion led to dramatic increases in solubility and permeability across both gastrointestinal and blood–brain barriers, which translated into significantly enhanced antioxidant and anti-butyrylcholinesterase activities [47].

Several limitations of the present study should be acknowledged. The study was conducted using in vitro models, including dissolution, permeability, and biological activity assays. While these methods offer valuable mechanistic insights, they cannot fully reproduce the complexity of in vivo conditions, particularly with regard to metabolism, distribution, and clearance. Moreover, in vitro biological activity assays could be expanded to include kinetic analyses. Future research should encompass comprehensive in vivo studies, combining pharmacokinetic evaluation with biological activity assessment in relevant animal models, to determine whether the improvements observed in vitro translate into enhanced bioavailability and therapeutic efficacy. Such investigations would form a continuation of the present work and allow for a more complete assessment of the translational potential of the developed delivery system.

## 4. Materials and Methods

### 4.1. Materials

AP (purity > 95%) was purchased from Xi’an Tian guangyuan Biotech Co. (Xi’an, China). AP Pharmaceutical Secondary Standard, Polyethylene glycol 6000 (PEG 6000), Poly(1-vinylpyrrolidone-co-vinyl acetate) (PVP-co-vinyl acetate), Poly(ethylene oxide), 2-hydroxypropyl-γ-cyclodextrin (HP-γ-CD) (0.5–0.7 molar substitution, Mw~1.580), 2-hydroxypropyl-α-cyclodextrin (HP-α-CD) (molar substitution 0.6, Mw~1.180), 2-hydroxypropyl-β-cyclodextrin (HP-β-CD) (molar substitution 0.8, Mw~1.460) were supplied by Sigma-Aldrich (Poznan, Poland). Polyvinyl caprolactam–polyvinyl acetate–polyethylene glycol graft copolymer (Soluplus^®^, SOL), and Polyvinylpyrrolidone K30 (Kollidon 30), vinylpyrrolidone–vinyl acetate copolymer (Kollidon VA64) were supplied by BASF SE (Ludwigshafen, Germany). Magnesium aluminometasilicate (Neusilin US2) was kindly provided by Fuji Chemical Industry (Minato, Tokyo, Japan). Polyvinyl alcohol (86–89% hydrolyzed) was obtained from Thermo Scientific, Kandel, Germany. Hydroxypropyl methylcellulose (HPMC) was provided by Shin-Etsu Chemical (Tokyo, Japan). Polyvinylpyrrolidone K25 (Vivapharm PVP K25F) was kindly supplied by JRS Pharma, Rosenberg, Germany. Prisma HT, along with the GIT lipid solution and acceptor sink buffer, was obtained from Pion Inc. (Forest Row, East Sussex, UK). Hydrochloric acid, sodium chloride, dimethyl sulfoxide (DMSO), and potassium dihydrogen phosphate were supplied by Avantor Performance Materials (Gliwice, Poland). High-performance liquid chromatography (HPLC) grade acetonitrile was sourced from Merck (Darmstadt, Germany). Formic acid with a purity range of 98–100% was supplied by POCH (Gliwice, Poland). Neocuproine, ammonium acetate, cupric chloride dihydrate, 2,2-Diphenyl-1-picrylhydrazyl (DPPH), 2,2′-azino-bis(3-ethylbenzothiazoline-6-sulfonic acid) (ABTS), acetic acid (99.5%), ethanol (96%), and Trolox, acetylcholine iodide, butyrylcholine iodide, acetylcholinesterase (AChE), butyrylcholinesterase (BChE), and 5,5′-dithiobis-(2-nitrobenzoic acid) (DTNB), Trizma^®^ base and Trizma^®^ hydrochloride were purchased from Sigma-Aldrich (Schnelldorf, Germany).

### 4.2. Preparation of the Systems and Solubility Studies

The systems were prepared with solubility enhancers. Hydrophilic polymers were selected for their ability to improve solubility through hydrogen bonding and enhance water affinity [110]. Cyclodextrins were included due to their known ability to form inclusion complexes with poorly soluble compounds, enhancing their solubility [111]. Copolymers like SOL were selected for their potential to improve solubility through micellar solubilization [112,113]. Other excipients, such as PVP and PEG, were included for their general use in pharmaceutical formulations to improve dissolution rates and stabilize the drug in solution [114,115]. Physical mixtures of the AP and selected solubility enhancers (SOL, Neusilin US2, PEG 6000, HPMC, Polyvinyl alcohol, PVP covinyl acetate, Vivapharm PVP K25F, Kollidon 30, Kollidon VA64, HP-α-CD, HP-β-CD, HP-γ-CD) were manually blended in a mortar for 5 min. A 10:90 (*w*/*w*) ratio of AP to excipient was selected based on preliminary screening studies, in which various AP concentrations (10–100%) were evaluated. The 10% AP formulation was chosen due to its ability to achieve an amorphous state, along with improved solubility. To assess the influence of solubility enhancers, 3.0 g of each AP–excipient mixture was placed in a supercritical fluid extraction vessel. For reasons of energy efficiency and in accordance with sustainability principles, the lowest effective temperature and pressure settings were chosen. Processing was conducted using an SFT-120 apparatus (Supercritical Fluid Technologies, Inc., Newark, DE, USA), where carbon dioxide was introduced to achieve a pressure of 5000 PSI (~345 bar). The static phase was maintained at 50 °C for 30 min.

Following preparation, the samples were withdrawn from the vessel and processed in a Tube Mill 100 control homogenizer (IKA, Warsaw, Poland) operating at 8000 rpm for 1 min. To evaluate the effectiveness of the solubilizing agents, a 24 h solubility assessment was carried out. For this test, an excess quantity of AP together with the formulated delivery systems was placed into glass vials containing 5 mL of distilled water. The vials were maintained in a MaxQ 4450 incubator (Thermo Scientific, Waltham, MA, USA) under constant agitation at 75 rpm and a controlled temperature of 25 °C for 24 h. After completion of the incubation, the resulting suspensions were filtered, and the filtrates were subjected to quantitative analysis using high-performance liquid chromatography (HPLC; analytical conditions described below).

Once SOL was identified as the excipient with the greatest potential for enhancing AP solubility, a comprehensive study of various amorphization parameters was carried out to maximize solubility enhancement. The following conditions were tested (Table 4):

The samples obtained during this step (AP–SOL 1–6) were subjected to the 24 h solubility study as described above, along with XRPD analysis (details provided below) to determine the optimal conditions.

### 4.3. X-Ray Powder Diffraction

The X-ray powder diffraction (XRPD) technique was used to investigate the crystalline or amorphous state. The diffraction patterns were acquired utilizing a PANalytical Empyrean diffractometer featuring CuKα radiation (wavelength of 1.54056 Å). Operational parameters included a tube voltage of 45 kV and a tube current of 40 mA. Measurement encompassed an angular range from 5° to 40° with a step size of 0.017° and a counting rate of 15 s/step. The crystalline structure of the AP was deposited with the Cambridge Crystallographic Data Centre (CCDC; deposition number: 2232365, https://www.ccdc.cam.ac.uk/, accessed on 31 July 2025) in Crystallographic Information File (CIF) format. A theoretical powder X-ray diffraction pattern was generated based on the CIF using Mercury software (version 2022.3.0, Build 390047). Analysis of the obtained data was conducted utilizing OriginPro 8 software (OriginLab Corporation, Northampton, MA, USA) [116].

### 4.4. Fourier-Transform Infrared Spectroscopy

Infrared spectra were recorded using an IRTracer-100 spectrophotometer (Kyoto, Kyoto Prefecture, Japan) equipped with an ATR (Attenuated Total Reflectance) accessory, operating in absorbance mode over the 4000–400 cm^−1^ frequency range. The measurements were taken at a resolution of 4 cm^−1^ with 400 scans and utilized Happ-Genzel apodization. To interpret the absorption bands corresponding to the analyzed compound (AP), a theoretical IR spectrum was generated using Density Functional Theory (DFT). Three-dimensional structure of AP (CID: 5280443) in sdf format was obtained from PubChem (https://pubchem.ncbi.nlm.nih.gov/, accessed on 8 April 2025). The calculations were carried out with the Gaussian 16C software package (Wallingford, CT, USA). Geometry optimization was performed using the B3LYP functional with the 6-311G(d,p) basis set. Gaussian 16C was run using computational resources provided by the “Eagle” HPC cluster for researchers, a service offered by the Poznan Supercomputing and Networking Center (https://pcss.plcloud.pl/, accessed on 8 April 2025) [117]. GaussView (Wallingford, CT, USA, Version E01) was applied to identify the normal modes and generate the theoretical FT-IR spectrum for further analysis [118]. Final spectral data analysis was performed with Origin Pro 8 software (OriginLab Corporation, Northampton, MA, USA).

### 4.5. High-Performance Liquid Chromatography

**Figure 9:**
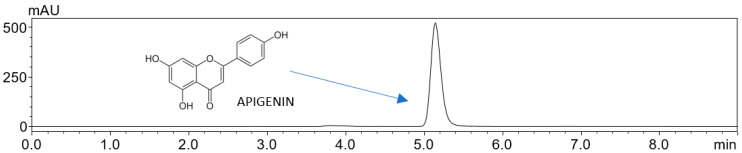
Chromatogram of apigenin.

Samples collected from the solubility, apparent solubility, and permeability experiments were examined using ultra-high-performance liquid chromatography coupled with a diode array detector (UHPLC-DAD, Shimadzu Corp., Kyoto, Japan) (Figure 9). The chromatographic separation employed a Dr. Maisch (Ammerbuch-Entringen, Germany) ReproSil Chiral-JM-R C18 column (150 mm × 4.6 mm, 5 µm) as the stationary phase. The mobile phase comprised acetonitrile and 0.1% formic acid in a 55:45 (*v*/*v*) ratio. Operating conditions included a column temperature of 30 °C, a 10.0 µL injection volume, and detection at 269 nm. The method produced a retention time of approximately 5.1 min, with a total run time of 9 min (validated, see Table S1). Data acquisition and analysis were performed using LabSolutions LC software (version 1.86 SP2, Shimadzu Corp., Kyoto, Japan).

### 4.6. The Dissolution-Rate Studies

The dissolution rate was determined using a paddle apparatus (Agilent Technologies, Santa Clara, CA, USA). AP (10.0 mg) and the corresponding physical mixture and delivery system with SOL (containing 10.0 mg of AP) were placed in gelatin capsules and fixed to springs to prevent floating. The test was conducted for 180 min in media with pH values of 1.2 (0.1 M HCl, simulating the gastric environment) and for 480 min in media with pH 6.8 (phosphate buffer, mimicking the intestinal environment). Each vessel contained 500 mL of medium, maintained at 37 °C, and rotated at 100 rpm. At specific time points, 1.0 mL samples were withdrawn and replaced with an equal volume of fresh medium at the same temperature. The samples were filtered through a 0.45 μm membrane filter before being analyzed by HPLC.

### 4.7. In Vitro Parallel Artificial Membrane Permeability Assay

Passive transport of AP across simulated biological barriers was evaluated using the parallel artificial membrane permeability assay (PAMPA). Separate tests were conducted for gastrointestinal tract (GIT) conditions at pH 1.2 and 6.8, and for the blood–brain barrier (BBB) environment at pH 7.4. The assay employed two stacked 96-well microfilter plates, one serving as the donor compartment (bottom) and the other as the acceptor compartment (top), separated by a membrane (Pion Inc., Billerica, MA, USA). AP delivery systems were first dissolved in dimethyl sulfoxide (DMSO) before being added to donor solutions adjusted to the target pH values. Incubation took place in a humidity-controlled chamber at 37 °C, lasting 3 h for GIT simulations and 4 h for BBB simulations. Following incubation, the compartments were separated, and AP concentrations were quantified via high-performance liquid chromatography with diode array detection (HPLC-DAD, Shimadzu Corp., Kyoto, Japan).

The apparent permeability coefficient (Papp) was calculated using the following equations:

(1) $$Papp=\frac{-ln\left(1-\frac{C_{A}}{C_{equilibrium}}\right)}{S\times \left(\frac{1}{V_{D}}+\frac{1}{V_{A}}\right)\times t}$$

where *V_D_*—donor volume, *V_A_*—acceptor volume, *C_equilibrium_*—equilibrium concentration, $C_{equilibrium}=\frac{C_{D}\times V_{D}+\;C_{A}\times V_{A}}{V_{D}+V_{A}}$, *S*—membrane area, *t*—incubation time (in seconds).

Compounds with a Papp value below 0.1 × 10^−6^ cm/s in the GIT model are classified as poorly permeable, those with a Papp value between 0.1 × 10^−6^ cm/s and 1 × 10^−6^ cm/s are considered moderately permeable, and those with a Papp value greater than 1 × 10^−6^ cm/s are highly permeable [82]. For the BBB model, substances with a Papp less than 2.0 × 10^−6^ cm/s are poorly permeable, those with Papp between 2.0 and 4.0 × 10^−6^ cm/s are of questionable permeability, and substances with Papp values above 4.0 × 10^−6^ cm/s are classified as highly permeable [119].

### 4.8. Stability Studies

After one year of storage under ambient conditions, the chemical stability of the AP delivery system with SOL was evaluated using HPLC (Shimadzu Corp., Kyoto, Japan). In parallel, the delivery system, which contained the AP in an amorphous form with SOL, was analyzed for any structural changes. XRPD analysis was performed to assess the solid-state properties and potential alterations in the amorphous form of the AP.

### 4.9. Antioxidant Activity

Three different assays were used to evaluate the antioxidant activity of the samples: DPPH, ABTS, and CUPRAC. Trolox was used as a reference in all assays. The studies were performed on samples after a 24 h dissolution study. The results are presented as the percentage of radical inhibition (or absorbance in the case of CUPRAC) and expressed as Trolox equivalents when they were measurable and did not reach the plateau region. Moreover, the ascending concentrations of AP in DMSO were prepared, the calibration curve was performed (presented in Figure S1 in Supplementary Materials), and IC_50_/IC_0_._5_ values were calculated. For the DPPH assay, a 96-well plate was used, and spectrophotometric analysis was performed [116]. A methanol solution of DPPH (0.2 mM) was used as the main reagent. For the assay, 25 µL of the AP, physical mixture, or delivery system was added to 175 µL of a DPPH solution. The reaction mixture was then kept at room temperature in darkness for 30 min. Absorbance readings at 517 nm were subsequently obtained using a Multiskan GO microplate reader (Thermo Fisher Scientific, Waltham, MA, USA). The blank, consisting of a mixture of DPPH solution and solvent, was measured simultaneously. DPPH scavenging activity was calculated using the following equation:

(2) $$A=\frac{A_{0}-A_{1}}{A_{0}}\times 100\%$$

where A_0_ is the absorbance of the control and A_1_ is the absorbance of the test sample. Each measurement was repeated six times.

In the ABTS assay, radicals were generated from ABTS by potassium persulfate, and antioxidants were measured based on their ability to reduce the ABTS•+ radical to its neutral form [120]. To carry out the assay, 200 µL of ABTS•+ solution and 10 µL of the AP/physical mixture/delivery system were added to 96-well plates. After incubation in the dark for 10 min at room temperature, absorbance was recorded at 734 nm using a Multiskan GO plate reader. The ABTS scavenging activity was calculated using the following formula:

(3) $$\mathrm{ABTS}\;\mathrm{scavenging}\;\mathrm{activity}\;(\%)\;=\frac{A_{0}-A_{1}}{A_{0}}\times 100\%$$

where A_0_ is the absorbance of the control and A_1_ is the absorbance of the sample.

For the CUPRAC assay, the reduction potential of the AP was determined [121]. This method involves the oxidation of phenolic compounds to quinones and the reduction of copper (II) ions. To perform the assay, 50 µL of the AP/physical mixture/delivery system and 150 µL of CUPRAC reagent were mixed and incubated in the dark for 30 min at room temperature. Absorbance was measured at 450 nm. All assays were conducted in six replicates.

### 4.10. Anticholinesterase Activity

The inhibitory potential of the AP against acetylcholinesterase (AChE) and butyrylcholinesterase (BChE) was evaluated using a spectrophotometric method based on a modified version of the Ellman assay [122] as previously described. This approach utilizes synthetic thiocholine derivatives as substrates. Upon enzymatic hydrolysis, thiocholine is released and subsequently reacts with 5,5′-dithiobis-(2-nitrobenzoic acid) (DTNB), generating the yellow-colored 3-carboxy-4-nitrothiolate anion (TNB), detectable at 405 nm. The percentage inhibition of enzyme activity was calculated using the formula:

(4) $$\mathrm{AChE}/\mathrm{BChE}\;\mathrm{inhibition}\;(\%)\;=\frac{1-(A_{1}-A_{1b})}{\left(A_{0}-A_{0b}\right)}\times 100\%$$

where A_1_ is the absorbance of the test sample, A_1b_ is the absorbance of the blank of the test sample, A_0_ is the absorbance of the control, and A_0b_ is the absorbance of the blank of the control.

### 4.11. Statistical Analysis

All statistical evaluations were conducted using Statistica version 13.3 (StatSoft, Krakow, Poland). Results are expressed as mean values accompanied by their respective standard deviations. The Shapiro–Wilk test was applied to assess the normality of data distributions, while Levene’s test was used to verify homogeneity of variances across groups. To identify statistically meaningful differences, a one-way analysis of variance (ANOVA) was employed, followed by the Bonferroni post hoc test, or if the assumptions of ANOVA were not met, the Kruskal–Wallis test was used instead (to compare the experimental results acquired for AP, the physical mixture, and in the system). A *p*-value of less than 0.05 was regarded as indicative of statistical significance.

## 5. Conclusions

This study demonstrates a stable and effective delivery system for apigenin (AP), which was developed using supercritical carbon dioxide processing with Soluplus^®^ (SOL) as a solubility enhancer. The resulting amorphous solid dispersion significantly improved AP aqueous solubility, from <1 μg/mL to over 8000 μg/mL, representing an over 8000-fold enhancement. XRPD and FTIR analyses confirmed the successful amorphization and suggested the presence of stabilizing intermolecular interactions between AP and the polymer matrix, contributing to both enhanced solubility and long-term physical and chemical stability over 12 months. The improved solubility of the AP–SOL system correlated with its markedly increased antioxidant potential and enhanced inhibition of acetylcholinesterase and butyrylcholinesterase, compared to pure AP and its physical mixture. Furthermore, PAMPA demonstrated significantly improved permeability through both gastrointestinal and blood–brain barrier models, supporting the system’s potential for enhanced oral and central nervous system bioavailability. The findings confirm that supercritical carbon dioxide processing is a promising and sustainable technique for generating high-performance amorphous solid dispersions of poorly soluble bioactive compounds like AP. The developed system not only improves solubility, dissolution rate, and biological performance but also exhibits excellent physicochemical stability, making it a strong candidate for future pharmaceutical and nutraceutical applications. However, pharmacokinetic studies are necessary to confirm whether the improvements observed in vitro translate into enhanced bioavailability in vivo.

## Figures and Tables

**Table 1:** Solubility of apigenin in the systems with various carriers. Mean values within a column followed by different superscript letters (a, b) are significantly different (*p* < 0.05), as indicated by the Compact Letter Display.

Compound	Carrier	Solubility (μg/mL)
Apigenin	-	<1 ^b^
	Soluplus (SOL)	6455.4 ± 27.2 ^a^
	Neusilin US2	1.3 ± 0.1 ^b^
	PEG 6000	1.5 ± 0.1 ^b^
	HPMC	10.4 ± 0.4 ^b^
	Polyvinyl alcohol	4.8 ± 0.3 ^b^
	PVP covinyl acetate	5.2 ± 0.2 ^b^
	Vivapharm PVP K25F	1.4 ± 0.1 ^b^
	Kollidon 30	<1 ^b^
	Kollidon VA64	9.6 ± 0.4 ^b^
	HP-α-CD	<1 ^b^
	HP-β-CD	1.0 ± 0.1 ^b^
	HP-γ-CD	2.9 ± 0.2 ^b^

**Table 2:** Selected characteristic bonds (in cm^–1^) of apigenin (AP), Soluplus (Sol), and the system of apigenin–Soluplus (AP–Sol system). Assignments of apigenin and Soluplus bands based on the literature [64,70,71]. The band assignment might also be accomplished using DFT computational tools for the partially amorphous phase or by employing the cluster approach [72,73].

AP	Sol [cm^−1^]	AP–Sol System [cm^−1^]	Band Assignment
428		↓	
453		↓	δCCC
473		*	δCOC + δCCC + δOCC
501		↓	γCCOC + γOCCC
579		↓	γOCCC
633		↓	γHOC + τHCCC
669		↓	γOCCC
692		↓	δCCC + γOCCC
737		↓	τHCCC + γOCCC
806		↓	τHCCC
827		↓	τCCCC
908		↓	τHCCC + τCCCC
	1018	1022	no information in the literature
	1053	1049	no information in the literature
	1148	1152	no information in the literature
1177		1182, ↓	δHOC + δHCC
	1196	↓	no information in the literature
1221		*	νCC + δHOC + δHCC
	1234	1236	νCOC in the ether groups
1244		*	δHCC + νCC
1269		*	νOC + δHCC
	1352	1354	no information in the literature
	1369	1364	O(C)O or NH
1400		*	δHOC + δHCC
1495		1503, ↓	δHCC + νCC
1557		↓	νCC + δHOC
1587		*	νCC + νOC
1605		*	νOC + νCC
1651		*	νOC + νCC
	2862	2859	νCH

Legend: ν—stretching vibrations; δ—in-plane bending vibrations; γ—out-of-plane bending vibrations; τ—torsional vibrations, ↓ indicates a decrease in intensity, and * means that the peak was not observed in the system and observed in the physical mixture.

**Table 3:** Antioxidant activity of AP, physical mixture (ph.m.), and the delivery system with Soluplus (SOL) determined by DPPH, ABTS, and CUPRAC Assays. Mean values within a column followed by different superscript letters (a–c) are significantly different (*p* < 0.05), as indicated by the Compact Letter Display.

	DPPH	ABTS	CUPRAC
AP	<0.1% ^c^	<0.1% ^b^	<0.01 ^c^
AP–SOL ph.m.	12.3 ± 0.9% ^b^	85.2 ± 2.4% ^a^	0.51 ± 0.03 ^b^
AP–SOL system	27.9 ± 0.7% ^a^	84.9 ± 3.1% ^a^	>1.00 ^a^
IC_50_/IC_0.5_	27.7 ± 0.6 mg/mL	59.0 ± 3.1 μg/mL	0.91 ± 0.02 mg/mL
Trolox IC_50_/IC_0.5_	92.04 ± 1.37 μg/mL	118.72 ± 3.87 μg/mL	56.15 ± 0.79 μg/mL

**Table 4:** Processing conditions for apigenin (AP) and Soluplus (SOL) delivery systems preparation at varying temperatures and pressures.

Name	Temperature (°C)	Pressure (PSI)
AP–SOL-1	50	5000
AP–SOL-2	65	5000
AP–SOL-3	80	5000
AP–SOL-4	50	6500
AP–SOL-5	65	6500
AP–SOL-6	80	6500

## Data Availability

Data are available in a publicly accessible repository. CCDC deposition number 2232365 contains the supplementary crystallographic data for this paper. These data can be obtained free of charge from The Cambridge Crystallographic Data Centre via www.ccdc.cam.ac.uk/structures (accessed on 31 July 2025).

## Supplementary Materials

The following supporting information can be downloaded at: https://www.mdpi.com/article/10.3390/ijms26178126/s1.

## Abbreviations

Abbreviation	Definition
ABTS	2,2′-azino-bis(3-ethylbenzothiazoline-6-sulfonic acid)
Aβ	Amyloid-β
AChE	Acetylcholinesterase
AMPK	AMP-activated protein kinase
ANOVA	Analysis of Variance
AP	Apigenin
ATR-FT-IR/FT-IR	(Attenuated Total Reflectance) Fourier-Transform Infrared (Spectroscopy)
BBB	Blood–Brain Barrier
BChE	Butyrylcholinesterase
CCDC	Cambridge Crystallographic Data Centre
CIF	Crystallographic Information File
CUPRAC	Cupric (ion) Reducing Antioxidant Capacity
DFT	Density Functional Theory
DMSO	Dimethyl Sulfoxide
DPPH	2,2-Diphenyl-1-picrylhydrazyl
GIT	Gastrointestinal Tract
HPLC-DAD/UHPLC-DAD	(Ultra-)High-Performance Liquid Chromatography with Diode Array Detector
HP-α-CD	2-Hydroxypropyl-α-cyclodextrin
HP-β-CD	2-Hydroxypropyl-β-cyclodextrin
HP-γ-CD	2-Hydroxypropyl-γ-cyclodextrin
HPMC	Hydroxypropyl Methylcellulose
Kollidon 30	Polyvinylpyrrolidone K30
Kollidon VA64	Vinylpyrrolidone–Vinyl Acetate Copolymer
Neusilin US2	Magnesium aluminometasilicate
ORAC	Oxygen Radical Absorbance Capacity
PAMPA	Parallel Artificial Membrane Permeability Assay
Papp	Apparent Permeability Coefficient
PEG 6000	Polyethylene Glycol 6000
PVA	Polyvinyl Alcohol
PVP-co-vinyl acetate	Poly(1-vinylpyrrolidone-co-vinyl acetate)
PVP K25F	Polyvinylpyrrolidone K25
ROS	Reactive Oxygen Species
SASP	Senescence-Associated Secretory Phenotype
scCO_2_	Supercritical Carbon Dioxide
SOL	Soluplus^®^ (polyvinyl caprolactam–polyvinyl acetate–polyethylene glycol graft copolymer)
TRKB	Tropomyosin receptor kinase B
XRPD	X-ray Powder Diffraction
