# Supplementary material for: Effective and Stable Senomorphic Apigenin Delivery System Obtained by Supercritical Carbon Dioxide Processing
Source: Int J Mol Sci. 2025 Aug 22;26(17):8126. doi: 10.3390/ijms26178126 (PMC12428233; doi:10.3390/ijms26178126)
Supplement: Supplementary file 1 [file ijms-26-08126-s001.zip › ijms-3794111-supplementary.pdf]

Supplementary materials

Table S1. HPLC method validation parameters

**Parameter**	**AP**
calibration curve	y= 76447123.21x – 10022.39
Range of linearity (μg mL^{-1})	1 – 100
Correlation coefficient (r)	0.999
Limit of detection (LOD): LOD = 3 SD/a (μg mL^{-1})	0.144
Limit of quantification (LOQ): LOQ = 10 SD/a (μg mL^{-1})	0.436
Intra-day precision, RSD (%)	
Small concentration	0.45
Medium concentration	0.31
High concentration	0.78
Inter-day precision, RSD (%)	
Small concentration	0.51
Medium concentration	0.44
High concentration	0.63
Accuracy (%)	99.52

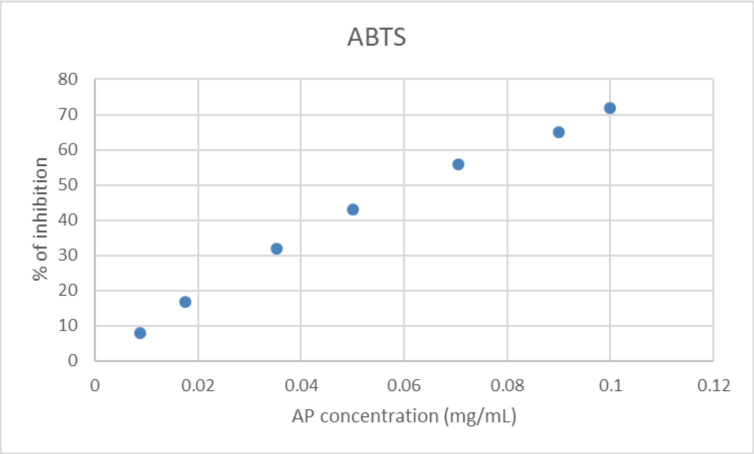

a)

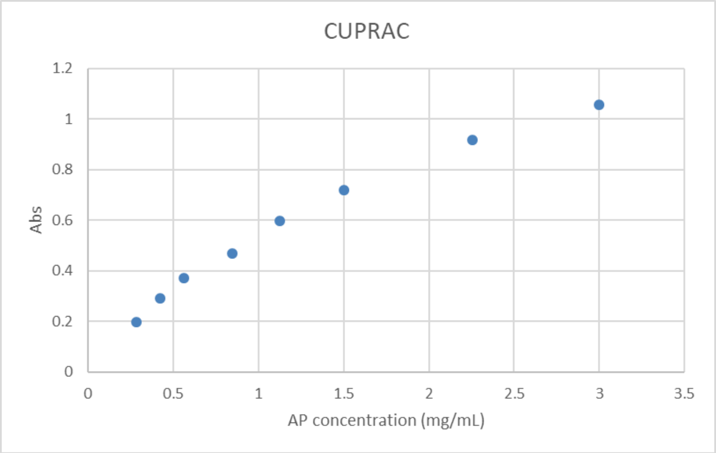

b)

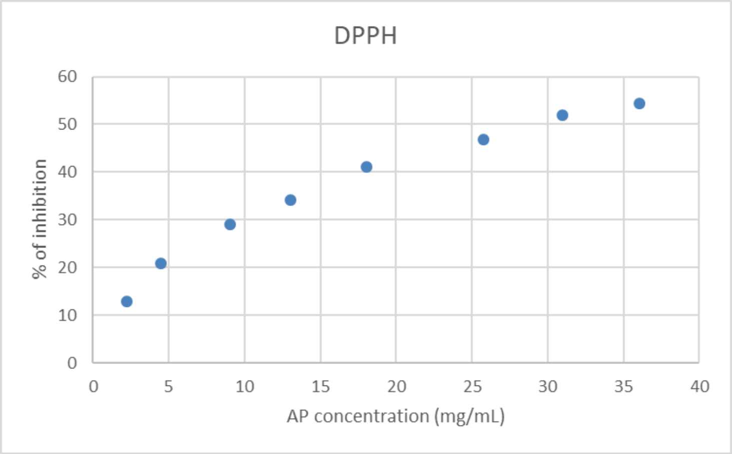

c)

Figure S1. AP concentration-dependent graphs for ABTS (a), DPPH (b), CUPRAC (c).
